# CARD9 gene silencing with siRNA protects rats against severe acute pancreatitis: CARD9‐dependent NF‐κB and P38MAPKs pathway

**DOI:** 10.1111/jcmm.13040

**Published:** 2016-12-13

**Authors:** Zhi‐wen Yang, Xiao‐xiao Meng, Chun Zhang, Ping Xu

**Affiliations:** ^1^ Songjiang Hospital Affiliated Shanghai First People's HospitalShanghai Jiao Tong UniversityShanghaiChina; ^2^ Shanghai Songjiang Hospital Affiliated to Nanjing Medical UniversityNanjingChina; ^3^ Shanghai First People's HospitalShanghai Jiao Tong UniversityShanghaiChina

**Keywords:** CARD9, siRNA *in vivo*, SAP, NF‐κB, P38MAPKs

## Abstract

We previously reported the up‐regulation of caspase recruitment domain 9 (*CARD9*) expressions in severe acute pancreatitis (SAP) patients, but little is known about its regulation. In this study, small interfering RNA (siRNA) was used to reduce the levels of CARD9 expression in sodium taurocholate‐stimulated SAP rats. CARD9 was overexpressed in SAP rats, which correlated with the severity of pancreatitis. When compared to the untreated group, the cohort that received the siRNA treatment demonstrated a significant reduction in pancreatic injury, neutrophil infiltration, myeloperoxidase activity and pro‐inflammatory cytokines. Furthermore, siRNAs showed that the reduction of CARD9 in SAP rats down‐regulated the expression of NF‐κBp65 and P38MAPK which are involved in the transcription and release of a wide variety of inflammatory cytokines. These findings provide evidence that CARD9 is up‐regulated in SAP rats and acts as a potential therapeutic target for the treatment thereof. Blocking the activation of NF‐κB and P38MAPK *via* siRNA‐mediated gene knock‐down of CARD9 appears to reduce the inflammatory response in pancreatic tissue.

## Introduction

SAP is characterized as an autodigestive and progressive inflammatory condition with a high mortality rate that ranges from 10% to 30%. No intervention strategies exist, and effective treatments have not been developed due to the insufficient understanding of SAP pathogenesis.

CARD9 belongs to a family of CARD‐containing adaptors, consisting of a carboxy‐terminal coiled‐coil domain and an amino‐terminal caspase recruitment domain 1. It is regarded as the signalling factor for downstream pattern recognition receptors, acting as a key adaptor in innate immune system signalling. Several studies demonstrate that CARD9 plays an important role against bacterial and fungal infection through inducing pivotal cytokine release and triggering innate immune system cells 2, 3, 4. However, under the conditions of sterile inflammation, the role of CARD9 expression is still unclear. To the best of our knowledge, the early course of SAP is characterized by non‐infectious inflammation. This is therefore the first report to demonstrate that CARD9 expression is up‐regulated in aseptic SAP and corresponds to previous findings 5. In this study, it was found that CARD9 is overexpressed in SAP patients and was identified as a potential molecular marker due to its close correlation with the outcome and severity of pancreatic injury in SAP patients 5. It has previously been shown that an up‐regulation of CARD9 expression in SAP patients is associated with an upstream activation of the NF‐κB and P38MAPK pathways 5. It has previously been reported that NF‐κB and P38MAPK is responsible for regulating the transcription of a wide variety of genes involved in the development of SAP 6, 7. Thus, we have suggested that the inhibition of CARD9 expression could serve as an effective treatment target for SAP through a novel molecular mechanism involving the CARD9‐dependent NF‐κB and P38MAPK pathways.

In this study and through the use of sodium taurocholate‐induced SAP, it was established that CARD9 was overexpressed in the rat model. Subsequently, the therapeutic effects and potential mechanisms for an inflammatory response in SAP rats were investigated through siRNA silencing of the *CARD9* gene.

## Materials and methods

### Animal model of SAP

Male Sprague‐Dawley rats weighing between 220 g and 280 g were obtained from the Chinese Academy of Science's Experimental Animal Center (Shanghai, China; Animal permit number: SYXK (Hu) 2009‐0086). All animals received standard animal care over a period of a week before experimentation took place. Experiments were conducted in rooms maintained at 23 ± 0.5°C and 50 ± 0.5% humidity with 12 hrs light/dark cycles. Rats were randomly divided into six groups. The rats were deprived of food, but were allowed access to water for a period of 12 hrs before harvesting the pancreatic tissue. Using a perfusion pump, the induction of SAP rats was achieved by retrograde perfusion of 5% sodium taurocholate in a volume–weight ratio of 1.5 ml/kg. Control rats received an intraperitoneal saline solution injection. All procedures were conducted according to the guidelines established by the animal ethical committee of Shanghai Jiao Tong University (China). Ascites volume, myeloperoxidase (MPO) activity and histopathological examinations were performed to evaluate the severity of the pancreatitis.

### Transfection with siRNA

Adenoviral constructs carrying siRNA against CARD9 were designed and produced by Obio Technology Co., Ltd (Shanghai, China). The siRNA sequences were inserted into the pAdeno‐U6‐CMV‐EGFP vector, and recombinant siRNA plasmids were transfected into 293T cells (ATCC, Manassas, VA, USA). The target sequences were 5′‐GGGTAAGCTACACAGGAAT‐3′. Sprague‐Dawley rats were injected intravenously in the tail with 1 × 10^9^ PFUs of CARD9 siRNA. At different predetermined time‐points (24, 48 and 72 hrs), the rats were killed after an intraperitoneal injection of pentobarbital (50 mg/kg). After exsanguination, the pancreatic tissue was collected for subsequent assays.

### *In vivo* studies

The rat models were randomly divided into six groups (*n* = 6 for each group). In the first group (control group), the Sprague‐Dawley rats were viewed as the control group and received an intraperitoneal injection of saline solution. In the second group (control siRNA group), normal Sprague‐Dawley rats were intravenously injected in the tail with 1 × 10^9^ PFU of control siRNA. The rats in the third group (CARD9 siRNA group) were intravenously injected in the tail with 1 × 10^9^ PFU of CARD9 siRNA. Using a perfusion pump, rats in the fourth group (SAP group) were induced by retrograde perfusion of 5% sodium taurocholate in a volume–weight ratio of 1.5 ml/kg. The fifth group of rats (control siRNA+SAP group) was intravenously injected in the tail with control siRNA before being induced in the same manner as the SAP group. The sixth and final groups (CARD9 siRNA+SAP) were intravenously injected in the tail with CARD9 siRNA before being induced in the same manner as the SAP group. Following each group's treatment, rats were free to drink water, but food consumption was not allowed.

### Ascite levels and serum amylase activity

At different predetermined time‐points, the rats were killed and ascites were collected from blood obtained directly from the aorta abdominalis. The amylase activity was examined by an automated biochemistry analyzer.

### ELISA

The rats were killed after an intraperitoneal injection of pentobarbital, and the blood was collected by puncturing the abdominal aorta. The serum was stored in a freezer at −80°C, and TNF‐α, IL‐6 and IL‐10 were measured using an ELISA assaying kit according to the manufacturer's instructions.

### Histological examinations

Pancreatic tissue was fixed with 10% formalin embedded in paraffin. Sections were stained with haematoxylin and eosin (H&E).

### Western blot

After treatment, pancreatic tissue was lysed with RIPA buffer, and the whole lysate quantified using the BCA protein assay kit (Beyotime Institute, Shanghai, China). Blot membranes were incubated overnight at 4°C with primary antibodies against P38MAPK, p‐P38MAPK, p‐NF‐κBp65 and NF‐κBp65 and subsequently incubated at room temperature for 2 hrs with the appropriate horseradish peroxidase‐conjugated secondary antibody (Abcam, Cambridge, MA, USA). The relative quantities of proteins were determined *via* scanning densitometry using a bio‐imaging analysis system (Bio‐Rad, Baltimore, MD, USA). Using the β‐actin protein as an endogenous control, the concentrations of other proteins were determined.

### Total RNA extraction and quantitative RT‐PCR

TRIzol Reagent (Invitrogen Carlsbad, CA, USA) was used to extract total RNA from pancreatic tissue of SAP rats. Then, real‐time PCR was applied to determine the level of CARD9, P38MAPK and NF‐κBp65 mRNA expression in the pancreatic tissue. The reaction mixture was amplified in a DNA thermal cycler (Thermo, Waltham, MA, USA), and the incubation and thermal cycling were under the following conditions: 95°C for 10 min., 40 cycles at 95°C for 15 sec. and 60°C for 45 sec.

The primers were as follows: β‐actin forward primer sequence 5′‐ACCACA GTCCATGCCATCAC‐3′, and reverse primer sequence 5′‐TCCACCACCCTGTTG CTGTA‐3′, p65 forward primer sequence 5′‐CATACGCTGACCCTA GCCTG‐3′, and reverse primer sequence 5′‐TTT CTTCAATCCGGTGGCGA‐3′, P38MAPK forward primer sequence 5′‐TAGACG AATGGA AGA GCCTGA‐3′, and reverse primer sequence 5′‐ACAGTGAAGTGGGATGGACAG‐3′, CARD9 forward primer sequence 5′‐TCTTTCGCAGACCCATGACA‐3′, and reverse primer sequence 5′‐GTCGTATTCCCGTGATCCCC‐3′.

### Statistical analysis

Data were presented as mean ± S.D. All experiments were independent of each other and repeated in triplicate. The statistical significance was assessed through a one‐way analysis of variance followed by SNK using SPSS13.0 software (SPSS, Inc., Chicago, IL, USA). All tests were two‐tailed, and statistical significance was defined as *P* < 0.05.

## Results

### Up‐regulation of CARD9 expression in SAP rats

**Figure 1:**
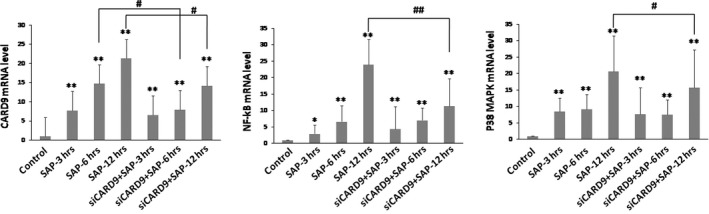
**CARD9 mRNA levels in SAP rats.** CARD9 mRNA, NF‐κB mRNA and P38MAPK mRNA from pancreatic tissue. *: SAP group or siCARD9 group vs. control group, *P* < 0.05; **: SAP group or siCARD9 group vs. control group, *P* < 0.01; #: SAP group vs. siCARD9 group, *P* < 0.05; ##: SAP group vs. siCARD9 group, *P* < 0.01.

**Figure 2:**
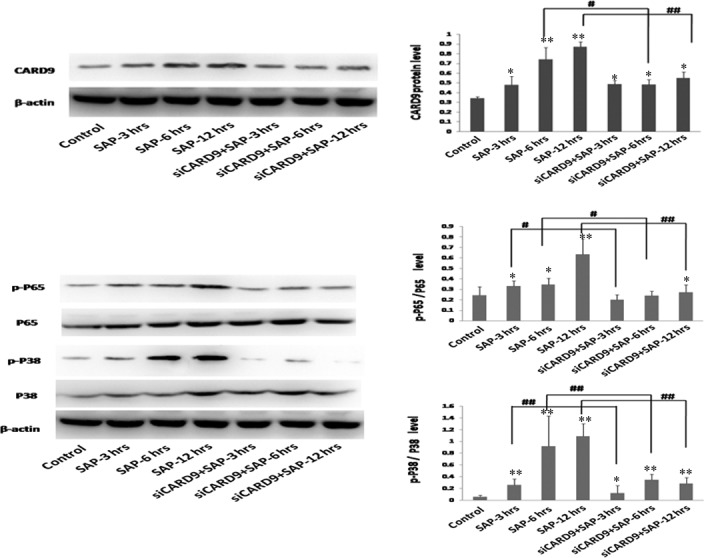
**CARD9 protein levels in SAP rats.** CARD9 protein, P‐p65/p65 protein and P‐p38/p38 protein from pancreatic tissue. *: SAP group or siCARD9 group vs. control group, *P* < 0.05; **: SAP group or siCARD9 group vs. control group, *P* < 0.01; #: SAP group vs. siCARD9 group, *P* < 0.05; ##: SAP group vs. siCARD9 group, *P* < 0.01.

A SAP rat model, using sodium taurocholate to induce a SAP state, was established in this study to determine the level of CARD9 expression in SAP incidents. Western blot and RT‐PCR methods were used to investigate the CARD9 expression at different time‐points. Figure 1 shows that CARD9 mRNA expression in the SAP group is higher than that of the control group (*P* < 0.05). Accompanied with the severity of pancreatitis, CARD9 mRNA in the SAP group significantly increased after three hours and reached a peak at 12 hrs. Up‐regulation of CARD9 expression was further confirmed by Western blot analysis (Fig 2). These data strongly indicated that CARD9 up‐regulation was positively correlated with the development of SAP.

### CARD9 siRNA knock‐down *in vivo*

**Figure 3:**
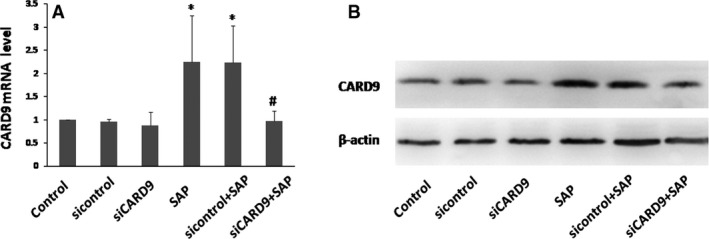
**CARD9 siRNA knockdown model *in vivo***. Pancreatic tissue was collected to assay *CARD9 mRNA* and protein levels at 48 h after adenoviral construct injection. *: SAP group or sicontrol group vs. control group, *P* < 0.05; #: SAP group vs. siCARD9 group, *P* < 0.05.

To investigate whether CARD9 expression could be an effective target in the treatment of SAP, rats were intravenously injected in the tail at 24, 48 and 72 hrs with adenoviral constructs carrying CARD9 siRNA. Then, RT‐PCR and Western blot analysis showed that there was a decrease in the level of CARD9 expression at 48 hrs after received the CARD9 siRNA. Control siRNA rats failed to reveal any differences in CARD9 expression in pancreatic tissue when compared to wild‐type rats (Fig 3). Furthermore, when compared to SAP rats, CARD9 siRNA‐treated SAP rats indicated a reduction of up to 60% of the targeted gene's mRNA expression (Fig 3). These data suggest that CARD9 gene silencing with siRNA was successfully used to establish an *in vivo* model for SAP.

### *In vivo* CARD9 siRNA alleviates pancreatitis severity, as well as lung and liver injury

Ascite volume and circulating plasma amylase analysis was performed to examine the therapeutic effect of *in vivo* CARD9 siRNA in SAP rats. As seen in Table 1, little ascites were observed in normal SD rats, while ascite volume was dramatically up‐regulated at 3 hrs in SAP rats and maintained at a high level at 6 and 12 hrs. After the *in vivo* siRNA gene knock‐down, ascite volume gradually declined at 3 hrs and reached a relatively low level at 6 and 12 hrs (*P* < 0.05). These data indicated that CARD9 gene silencing using siRNA causes a significant reduction in ascite volume when compared to the SAP group. In addition, serum amylase in the SAP group was significantly higher compared to the control group (*P* < 0.05). However, there was no difference in the levels of serum amylase in siRNA‐treated animals when compared to the SAP group. This might be attributed to the fact that the levels of serum amylase does not positively correlate with the severity of pancreatitis.

**Table 1:** siRNA‐treated SAP rats

Group	Ascites volume (ml)	Plasma amylase (U/l)	MPO(U/g)
Control	1.25 ± 0.27	1034.17 ± 113.1	1.61 ± 0.69
SAP‐3h	5.83 ± 1.17	1880.5 ± 423.62	3.59 ± 1.03^a^
SAP‐6h	7.5 ± 1.22^a^	2785.17 ± 337.78	4.59 ± 0.87^a^
SAP‐12h	7.5 ± 1.64^a^	3868.67 ± 523.35	9.65 ± 0.67^a^
siCARD9‐ SAP‐3h	5.66 ± 0.52	1964.5 ± 166.33	3.07 ± 0.78^a^
siCARD9‐ SAP‐6h	6.83 ± 1.33^a^	3021.17 ± 220.69	3.86 ± 0.53^a^, ^b^
siCARD9‐ SAP‐12h	6.83 ± 1.94^a^	3990.17 ± 169.17	5.43 ± 1.03^a^, ^b^

Control (wild‐type) and siCARD9 (siRNA CARD9) rats were compared. Values are expressed as the mean ± S.D., *n* = 6.

a SAP group or siCARD9 group *versus* control group, *P* < 0.05.

b SAP group *versus* siCARD9 group, *P* < 0.05. Ascite volume and MPO activity were associated with the severity of pancreatitis.

Myeloperoxidase activity in the pancreas was quantified as a marker of neutrophil sequestration in this organ. Table 1 shows that MPO activity in the SAP group significantly increased at 3 hrs and reached a peak at 12 hrs, while the CARD9 gene silencing with siRNA led to a significant decrease in pancreatic MPO activity (*P* < 0.05). Myeloperoxidase activity obviously suggests that CARD9 siRNA treatment could effectively alleviate the infiltration of leucocytes in the pancreas.

**Figure 4:**
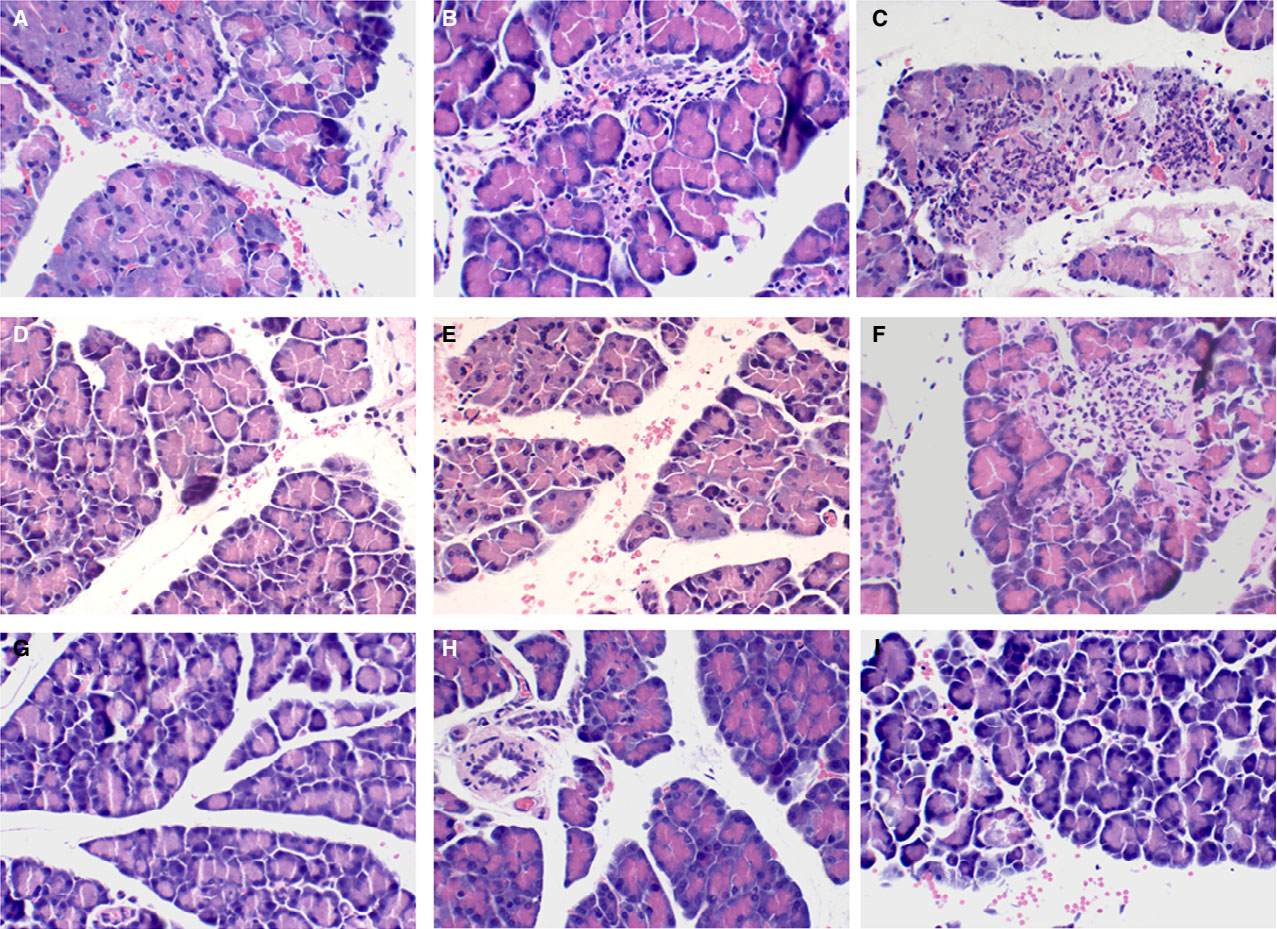
**Histological examination of the pancreas.** A: SAP group, observed at 3 h; B: SAP group, observed at 6 h; C: SAP group, observed at 12 h; D: siRNA group, observed at 3 h; E: siRNA group, observed at 6 h; F: siRNA group, observed at 12 h; G: sham‐operation group, observed at 3 h; H: sham‐operation group, observed at 6 h; I: sham‐operation group, observed at 12 h.

As shown in Figure 4, there were no remarkable pathological changes in control rats. In SAP rats, histological characterization found that interstitial oedema, haemorrhage, inflammatory cell infiltration and focal necrosis had occurred. Treatment of SAP rats with CARD9 siRNA at 6 and 12 hrs resulted in an obvious amelioration of pancreatic injury. Consistent with the above MPO activation, the number of infiltrating leucocytes found in the pancreas in the siRNA group was markedly decreased than those identified in the SAP group. Pancreatic pathology further confirmed that CARD9 siRNA could ameliorate the degree of inflammation in the pancreas.

**Figure 5:**
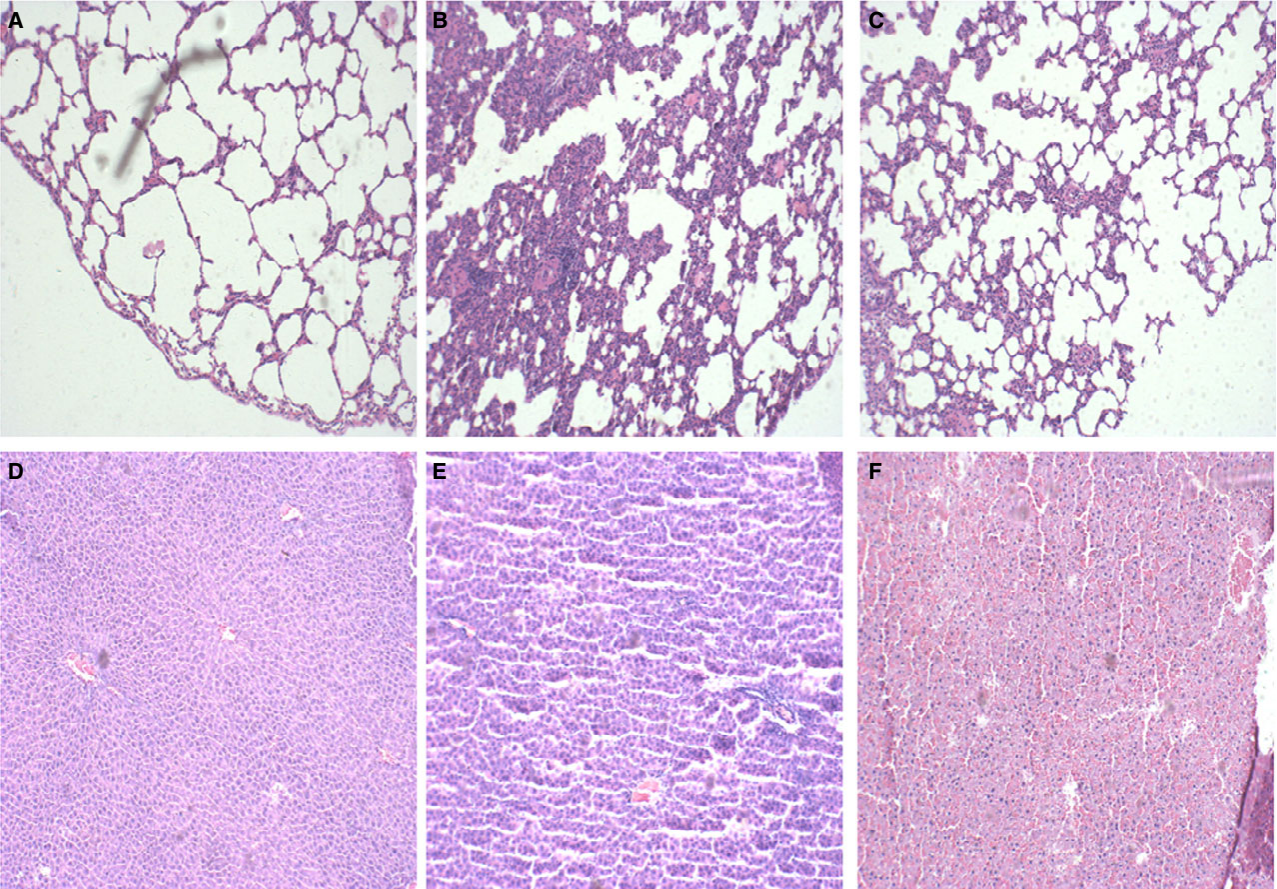
Histological examination of the lung and liver at 6 hrs. (**A–C**) lung tissues (**A**) control group; (**B**) SAP group; (**C**) siRNA group. (**D–F**) liver tissues (**D**) control group; (**E**) SAP group; (**F**) siRNA group.

SAP rats receiving CARD9 siRNA therapy showed an obvious inhibition of inflammatory cell infiltration, leading to an amelioration of lung injury. The same results were found in liver tissue (Fig. 5).

### *In vivo* CARD9 siRNA inhibits inflammatory cytokines and down‐regulates TLR4 and Dectin1 receptors

**Figure 6:**
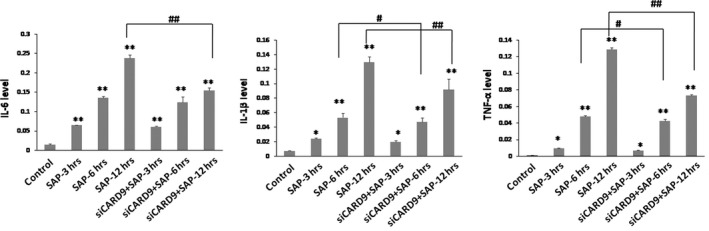
**Level of cytokines in serum samples.** The protein levels of IL‐6, IL‐1β and TNF‐α were determined by ELISA. control group(wild‐type rats), siCARD9 group (siRNA CARD9). Values were expressed as the mean ± SD, *n* = 6. *: SAP group or siCARD9 group vs. control group, *P* < 0.05; **: SAP group or siCARD9 group vs. control group, *P* < 0.01; #: SAP group vs. siCARD9 group, *P* < 0.05; ##: SAP group vs. siCARD9 group, *P* < 0.01.

**Figure 7:**
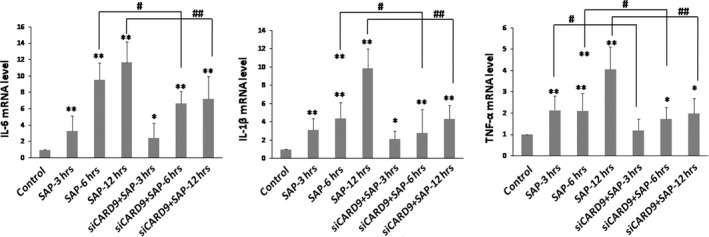
**Level of cytokines in pancreatic tissue.** The mRNA levels of *IL‐6*,* IL‐1β* and *TNF‐α* were determined by quantitative RT‐PCR. control group(wild‐type rats), siCARD9 group (siRNA CARD9). Values were expressed as the mean ± SD, n = 6. *: SAP group or siCARD9 group vs. control group, *P* < 0.05; **: SAP group or siCARD9 group vs. control group, *P* < 0.01; #: SAP group vs. siCARD9 group, *P* < 0.05; ##: SAP group vs. siCARD9 group, *P* < 0.01.

**Figure 8:**
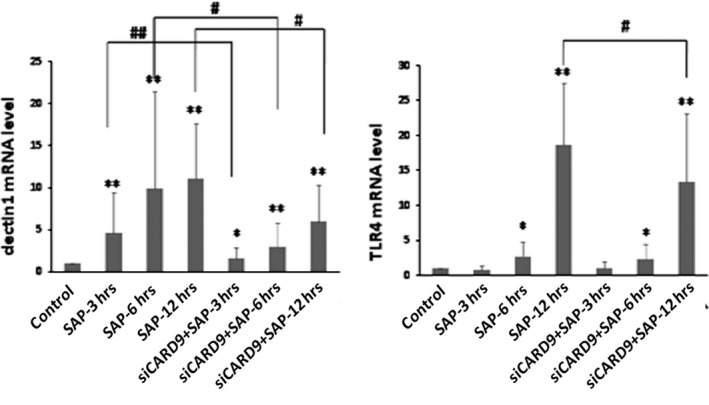
**The mRNA levels of *TLR4* and *Dectin1* in pancreatic tissue.** control group(wild‐type rats), siCARD9 group (siRNA CARD9). Values were expressed as the mean ± SD, *n* = 6. *: SAP group or siCARD9 group vs. control group, *P* < 0.05; **: SAP group or siCARD9 group vs. control group, *P* < 0.01; #: SAP group vs. siCARD9 group, *P* < 0.05; ##: SAP group vs. siCARD9 group, *P* < 0.01.

**Figure 9:**
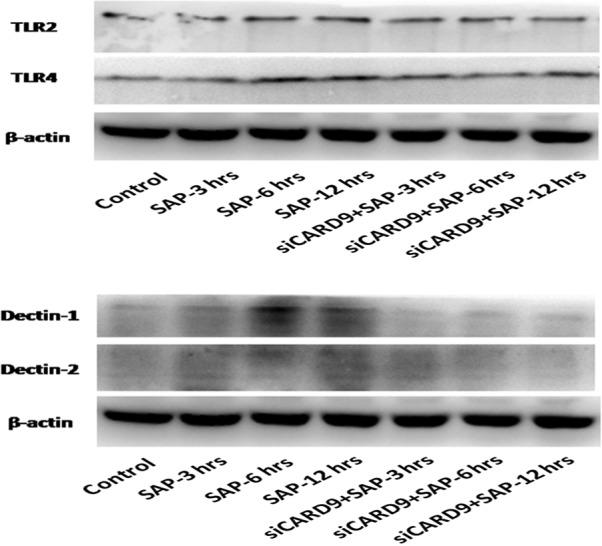
**The protein levels of TLR4 and Dectin1 in pancreatic tissue.** control group(wild‐type rats), siCARD9 group(siRNA CARD9).

As shown in Figure 6, the serum levels of TNF‐α, IL‐6 and IL‐1β increased significantly at 3 hrs in the SAP group. The serum levels of these inflammatory cytokines and the degree of pancreatic damage were found to be related to the severity of the inflammatory response in SAP rats. After treatment with CARD9 siRNA, there was a significant inhibitory effect on the levels of TNF‐α, IL‐6 and IL‐1β expression when compared to the SAP group. Consistent with serum levels, these inflammatory cytokines showed similar changes in the pancreatic tissue (Fig. 7). Accompanied by the noticeable and decreased production of TNF‐α, IL‐6 and IL‐1β in CARD9 siRNA knock‐down rats, the TLR4 and Dectin1 receptors appeared to show a similar down‐regulation in pancreatic tissue (Figs 8 and 9). Thereby, CARD9 might serve as a novel and effective therapeutic target for SAP.

### Reduction of CARD9 down‐regulates NF‐κB and P38MAPK signalling in SAP rats

NF‐κB and P38MAPK signalling pathways have been shown to play an important role in the development of SAP, inducing the release of several inflammatory cytokines. To address the role that CARD9 up‐regulation plays in NF‐κB and P38MAPK signal activation, siRNA was used to deplete CARD9 expression in sodium taurocholate‐stimulated SAP rats. As seen in Figure 1, CARD9 mRNA levels gradually increased and reached a peak at 12 hrs, which was consistent with the NF‐κB and P38MAPK signalling activation in SAP rats. After the *in vivo* CARD9 gene silencing with siRNA, *NF‐κB* mRNA and *P38MAPK* mRNA levels significantly decreased in siRNA‐treated rats at 12 hrs and were accompanied by reduced CARD9 mRNA. As seen in Figure 2, CARD9, NF‐κBp65 and P38MAPK expression at protein level were markedly increased in the pancreatic tissue of SAP rats. After *in vivo* CARD9 gene silencing with siRNA, the level of unphosphorylated and phosphorylated *NF‐κBp65* and *P38MAPK* gene regions was also decreased in siRNA‐treated group at 3, 6 and 12 hrs (*P* < 0.05). Therefore, these data obviously address the potential mechanisms that the CARD9 protein holds as a co‐activator in inducing the NF‐κB and P38MAPK signalling pathways in SAP rats.

## Discussion

Along with the advances in molecular biology techniques, the pathogenesis of inflammatory cytokines inducing SAP is becoming increasingly more complex 8. It is currently believed that the P38MAPK and NF‐κB pathways are the main pathways of pro‐inflammatory cytokines in SAP. They induce the release of inflammatory cytokines thereby initiating the inflammatory cascade. Consequently, this exacerbates the systemic inflammatory response syndrome which finally results in multiple organ dysfunction syndrome in SAP patients 6, 7. In addition, a recent study showed that NF‐κB and P38MAPK protected pancreatic cells from inflammation‐mediated apoptosis when associated with intra‐acinar trypsin activation 7, 9. Thus, the inhibition of NF‐κB and P38MAPK activity is likely to be a useful approach in alleviating the inflammatory response in SAP patients.

It has been previously found that CARD9 overexpression is involved in sterile‐induced inflammation 5. Of note, up‐regulation of CARD9 expression in SAP patients was associated with the upstream activation of molecules linked to the NF‐κB and P38MAPK pathways 5. Due to safety concerns, clinical studies are limited. It is therefore difficult to determine whether CARD9 may become an attractive target as a therapy for patients diagnosed with SAP. In this study, *in vivo* gene knock‐down models were used to further investigate the role of CARD9 in SAP rats. CARD9 expression was reduced using an *in vivo* adenoviral‐mediated transfer, and its effects on the secretion of inflammatory mediators in sodium taurocholate‐stimulated SAP rats were evaluated. Neutrophil infiltration and MPO activity in the pancreas were viewed as indicators of pancreatic injury 10. It was observed that *CARD9* gene silencing with siRNA protected rats against pancreatic injury as indicated by histopathological examination, leucocyte infiltration and MPO activity in the pancreas. Furthermore, TNF‐α, IL‐6 and IL‐1β expression has shown a close correlation with the infiltration of activated inflammatory cells into the pancreas and is also associated with the severity of pancreatitis. The blockage of these pro‐inflammatory cytokines by the administration of monoclonal antibodies can lead to a reduction of inflammatory cytokines and attenuation of acute pancreatitis 11. Interestingly, these findings also showed that the levels of TNF‐α, IL‐6 and IL‐1β were dramatically decreased in *CARD9* knockout rats. The reduced levels of pro‐inflammatory cytokines were consistent with the severity of pancreatic injury as demonstrated by histological examination and MPO activity. Therefore, the present study strongly suggests that CARD9‐targeted siRNA treatment of SAP rats holds promising therapeutic potential. The underlying mechanisms involved in a therapeutic effect of CARD9‐targeted siRNA will be analysed in subsequent experiments.

Several studies support the concept that CARD9 contributes to the innate immune system's response to infectious diseases through the recognition of bacterial, viral and fungal pathogens 12, 13, 14. A deficiency of CARD9 failed to trigger an innate host defence reaction against microbial infections. It is becoming obvious that CARD9 is mainly involved in non‐TLR signalling pathways by mediating Dectin1‐induced NF‐κB activation. The binding of β‐glucan to Dectin1 can initiate a direct recruitment of CARD9 resulting in NF‐κB activation, but not P38MAPK activation 15, 16, 17. CARD9 also induces TLR‐dependent signalling pathways by mediating TLR4‐induced P38MAPK activation. This signalling cascade however fails to activate the NF‐κB system 12. Although a general consensus concerning the role of CARD9 expression in infectious diseases has been reached, its role in sterile‐induced inflammation is still less obvious. Without any infections associated with micro‐organisms at the onset of disease, SAP has generally been regarded as being linked to sterile inflammation 18, 19. It has previously been found that CARD9, NF‐κB and P38MAPK expression is up‐regulated in SAP patients 5. These data strongly suggest a potential mechanism through which NF‐κB and P38MAPK molecules are activated by the up‐regulated expression of CARD9 in SAP. It was further found that CARD9 gene silencing with *in vivo* siRNA could significantly and simultaneously down‐regulate the expression level of NF‐κB and P38MAPK, providing strong evidence that CARD9 is involved in these signalling pathways. When considering the difference between infectious and sterile inflammation, CARD9 was found to be a co‐activator of NF‐κB and P38MAPK‐dependent target genes which include *TNF‐*α, *IL*‐6, and *IL*‐1β. These findings demonstrate that siRNA targeted *CARD9* gene silencing could have the anti‐inflammatory potential required for the treatment of SAP through the inhibition of the NF‐κB and P38MAPK pathways.

This *in vivo* gene knock‐down model has been shown to effectively down‐regulate gene expression through different delivery methods. RNA interference (RNAi) technology, as a unique gene knock‐down strategy, binds to complementary target mRNA thereby ear‐marking these specific sequences for degradation. RNA interference technology has demonstrated its ability to study protein expression and its function in preventing and/or treating disease 20, 21, 22. In addition, siRNA has been extensively tested as a potent therapy for inflammatory diseases in animal models 23, 24. Recently, many studies have indicated that an adenovirus‐mediated siRNA transfer method is a more efficient delivery system in decreasing the *in vivo* expression of pancreatic proteins 25, 26, 27. For example, siRNA‐mediated gene knock‐down of *Ins*2 mRNA was established when siRNA constructs were administered intravenously through the tail vein. A 33% reduction in expression of the target gene's mRNA was observed after animals received 100 μg of siRNA 25. In these studies, siRNA‐mediated gene knock‐down has developed into a mature method to localize the down‐regulation of target genes. However, siRNA transfer may have caused an incomplete knockout of the target genes in mice which resulted in a failure to knock‐down the genes required for investigating protein and gene function. In previous reports, CARD9^−/−^ mice were proposed as an animal model to maximize the efficacy of the gene knock‐down in anti‐inflammatory research 1, 28. Until now, the gene‐silencing effects of *in vivo* CARD9 RNAi were completely unknown. In the present study, siRNA of *CARD9* was transfected into SAP rats to explore its anti‐inflammatory effects. This study indicates that CARD9 siRNA is an effective method in transfecting the pancreas of rats and can be achieved through administrating siRNA through the tail vein for up to 2 days. More importantly, the administration of CARD9 siRNA has an obvious therapeutic effect on the pancreatic injury in sodium taurocholate‐stimulated SAP rats. These findings provide sufficient support to the hypothesis that CARD9 plays a key role in the development of SAP. Therefore, *in vivo* CARD9 RNAi gene‐silencing has been used to down‐regulate *CARD9* expression levels in SAP rats. This has not been observed in CARD9^−/−^ rats induced through clustered regularly interspaced short palindromic repeats (CRISPR/Cas9) and transcription activator‐like effector nuclease.

The adaptor molecule CARD9 is mainly expressed in monocytes/macrophages 29, lung 1 and the intestinal tract 28. In this study, it has also been demonstrated that CARD9 is expressed in the pancreas. It is currently accepted that monocyte/macrophage activation is an early event during in the course of SAP, followed by rapid systemic organ failure of the pancreas, intestinal tract and lungs. This therefore provides additional clues as to the potential involvement of CARD9 in the development of SAP inflammation.

In summary, this is the first report that siRNA silencing of *CARD9* provides protection against sodium taurocholate‐induced pancreatitis in rats. CARD9 also plays a key role in modulating the sterile inflammatory response through the NF‐κB and P38MAPK pathways.

## Disclosure statement

The authors have nothing to disclose.
